# The Impact of Domestication on Aboveground and Belowground Trait Responses to Nitrogen Fertilization in Wild and Cultivated Genotypes of Chickpea (*Cicer* sp.)

**DOI:** 10.3389/fgene.2020.576338

**Published:** 2020-12-02

**Authors:** Edward Marques, Christopher P. Krieg, Emmanuel Dacosta-Calheiros, Erika Bueno, Emily Sessa, R. Varma Penmetsa, Eric von Wettberg

**Affiliations:** ^1^Department of Plant and Soil Science and Gund Institute for the Environment, University of Vermont, Burlington, VT, United States; ^2^Department of Biological Sciences, Florida International University, Miami, FL, United States; ^3^Department of Biology, University of Florida, Gainesville, FL, United States; ^4^Department of Plant Sciences, University of California, Davis, Davis, CA, United States

**Keywords:** resource use efficiency, functional traits, cicer, roots, phenotypic plasticity 3

## Abstract

Despite the importance of crop responses to low fertility conditions, few studies have examined the extent to which domestication may have limited crop responses to low-fertility environments in aboveground and belowground traits. Moreover, studies that have addressed this topic have used a limited number of wild accessions, therefore overlooking the genotypic and phenotypic diversity of wild relatives. To examine how domestication has affected the response of aboveground and belowground agronomic traits, we measured root and leaf functional traits in an extensive set of wild and domesticated chickpea accessions grown in low and high nitrogen soil environments. Unlike previous studies, the wild accessions used in this study broadly capture the genetic and phenotypic diversity of domesticated chickpea’s (*Cicer arietinum*) closest compatible wild relative (*C. reticulatum*). Our results suggest that the domestication of chickpea led to greater capacities for plasticity in morphological and biomass related traits but may have lowered the capacity to modify physiological traits related to gas exchange. Wild chickpea displayed greater phenotypic plasticity for physiological traits including stomatal conductance, canopy level photosynthesis, leaf level photosynthesis, and leaf C/N ratio. In contrast to domesticated chickpea, wild chickpea displayed phenotypes consistent with water loss prevention, by exhibiting lower specific leaf area, stomatal conductance and maintaining efficient water-use. In addition to these general patterns, our results indicate that the domestication dampened the variation in response type to higher nitrogen environments for belowground and aboveground traits, which suggests reduced genetic diversity in current crop germplasm collections.

## Introduction

The practice of artificial selection was a critical innovation in human history that allowed for the rapid directional modification of traits in plants and animals. In crops like cereals and legumes, humans primarily selected for traits such as fruit indehiscence, reduced seed dormancy, and yield (e.g., Gross and Olsen, 2010; Meyer et al., 2012; Olsen and Wendel, 2013; Smýkal et al., 2018). However, the selection to modify one trait can often lead to a modification in other traits due to trait covariation and underlying genetic linkage (Lande and Arnold, 1983; Price and Langen, 1992). The evolution of traits through the inadvertent selection of correlated traits and genetic linkage are well documented in a variety of species (Rauw et al., 1998; Hoekstra et al., 2001; Kingsolver et al., 2001; Hereford et al., 2004; Kingsolver and Pfennig, 2007). In crops, similar patterns of correlated selection combined with population bottlenecks during domestication may have unintentionally altered non-target traits, potentially canalizing crop responses to different environmental conditions (Flatt, 2005; Morrell et al., 2011; Meyer et al., 2012; Gaut et al., 2018; Smýkal et al., 2018; Lye and Purugganan, 2019). Understanding the degree to which domestication has canalized or otherwise altered plant traits, and the ability of plants to respond to low fertility environments can aid agricultural programs to combat food insecurity in a changing global climate.

Although many comparative studies have demonstrated how artificial selection can lead to marked decreases in genomic and phenotypic variation in domesticated plants compared to wild relatives (e.g., Morrell et al., 2011; Olsen and Wendel, 2013; Gaut et al., 2018; Hufford et al., 2019; Lye and Purugganan, 2019), the majority of comparative research of phenotypes has focused on the impacts of domestication on aboveground agronomic traits such as seed size or shattering (e.g., Milla et al., 2015; Ogutcen et al., 2018; Smýkal et al., 2018). Relatively few studies have examined the potentially canalizing effects of domestication on belowground functional traits such as root architecture and root-soil-nutrient dynamics (e.g., Bulgarelli et al., 2015; Milla et al., 2015; Pérez-Jaramillo et al., 2016). Even fewer have taken a whole-plant approach to understand the impact of domestication on aboveground and belowground traits in tandem; thus, limiting our understanding of how domestication may have impacted plant function. Furthermore, most studies assessing the effects of domestication on crops have utilized very small numbers of genotypes of wild relatives (e.g., Warschefsky et al., 2014), limiting the power and potential to extrapolate from these comparisons.

Many crop wild relatives are found in environments with limited water availability and more nutrient-poor soils compared to their domesticated counterparts that occur in agricultural farms (e.g., Grossman and Rice, 2012; McKey et al., 2012; Milla et al., 2015). However, these wild habitats are heterogenous, and likely to maintain phenotypic plasticity, in contrast to trait canalization (*sensu* Flatt, 2005). As a result, a shift to fertile environments during domestication (i.e., as humans often initially cultivated richer valley soils, and learned to till soils and fertilize crops with animal waste) may have relaxed selective pressures on plant functional traits that impact resource acquisition like carbon, nitrogen, and water uptake from nutrient-poor soils or canalized responses under high fertility conditions (Grossman and Rice, 2012; Martin and Isaac, 2015). Canalization of nutrient uptake traits under nutrient-rich environments in cultivated crop lineages could lead to poorer performance than ancestral wild populations in nutrient-limiting environments from erosion of alleles for these traits in cultivated genepools. This would result in a reduced capacity to grow in low nutrient conditions, such as those typical in many small holder farming systems in the developing world, for farmers restricted to marginal soils and to some organic production systems.

The impacts of domestication on belowground traits may be particularly pronounced for crops with complex soil interactions such as legumes. A recent study suggests that the domestication of common bean (*Phaseolus vulgaris* L.), for which domestication and post-domestication selection by humans has focused largely on the bean itself (i.e., pod shattering, yield, size, and flavor), has also resulted in shifts in traits critical to soil interactions and nutrient dynamics including root microbiome composition, increased specific root length (SPL), and decreased root density (Pérez-Jaramillo et al., 2017). Despite the impact of domestication on agronomic traits, a broad set of root functional traits remain unexplored for most of the world’s most economically important crop species. For example, chickpea (*Cicer arietinum* L.) is the second most important grain legume globally and the leading legume in South Asia (FAO et al., 2017). The need for such studies in economically important crop species such as chickpea is more urgent than ever, with reductions in rainfall and soil fertility predicted to result in decreased yields in several food-insecure areas like India, Ethiopia, and Turkey, where chickpea is a key source of nutritional security and a cash crop (Singh et al., 2014; Ahmed et al., 2016). Therefore, understanding the degree to which domestication has impacted plant traits, and the ability of plants and traits to respond to new environments, is critical to adapting agricultural programs in a changing climate.

To understand how domestication affected aboveground and belowground agronomic traits, resource-use efficiency, and adaptive capacity in crops, we assembled a uniquely large collection of wild chickpeas from southeastern Turkey, providing sufficient numbers of genetically distinct wild genotypes to examine differentiation in aboveground and belowground phenotypes between cultivated crops and their wild relatives (von Wettberg et al., 2018). We grew wild and domesticated chickpea accessions in low and high nitrogen concentrations and measured root and leaf functional traits. We hypothesized that if domestication for typical agronomic traits has resulted in inadvertent selection in other functional traits due to cultivation in higher fertility environments that are typical of agriculture, then (1) wild accessions will have traits consistent with greater performance and resource use efficiency in low nutrient conditions compared to domesticated accessions and (2) domesticated accessions will exhibit lower phenotypic plasticity in root and leaf functional traits.

## Materials and Methods

### Plant Germplasm Used

Twenty-seven genetically diverse accessions of chickpea were used in this study (Table 1). Six accessions: CDC Frontier, ICC16207, Gokce, Dwelley, Myles, and UC15 are cultivars originating from four countries: United States, Canada, Turkey, and India. These accessions were selected because they represent both chickpea market types, Desi and Kabuli (Penmetsa et al., 2016) and are widely grown in their native countries. The remaining 21 accessions are wild chickpea lines systematically collected from different regions of Turkey, the native range of wild chickpea (von Wettberg et al., 2018). These accessions were selected to maximize genetic and native environmental differences in the material to capture as much wild diversity as possible.

**Table 1:** Germplasm used in the study

Germplasm	Species	Geographical origin	History	Market type
CDC Frontier	*Cicer arietinum*	Canada	Domesticated	Kabuli
ICC16207	*Cicer arietinum*	India	Domesticated	Desi
Gokce	*Cicer arietinum*	Syria	Domesticated	Kabuli
Dwelley	*Cicer arietinum*	United States	Domesticated	Kabuli
Myles	*Cicer arietinum*	United States	Domesticated	Kabuli
UC 15	*Cicer arietinum*	United States	Domesticated	Kabuli
Bari1 092	*Cicer reticulatum*	Turkey	Wild	Wild
Bari2 072	*Cicer reticulatum*	Turkey	Wild	Wild
Bari3 072n2	*Cicer reticulatum*	Turkey	Wild	Wild
Bari3 100	*Cicer reticulatum*	Turkey	Wild	Wild
Bari3 106	*Cicer reticulatum*	Turkey	Wild	Wild
Besev 075	*Cicer reticulatum*	Turkey	Wild	Wild
Besev 079	*Cicer reticulatum*	Turkey	Wild	Wild
CudiA 152	*Cicer reticulatum*	Turkey	Wild	Wild
CudiB 022C	*Cicer reticulatum*	Turkey	Wild	Wild
Derei 070	*Cicer reticulatum*	Turkey	Wild	Wild
Derei 072	*Cicer reticulatum*	Turkey	Wild	Wild
Egill 065	*Cicer reticulatum*	Turkey	Wild	Wild
Egill 073	*Cicer reticulatum*	Turkey	Wild	Wild
Kalka 064	*Cicer reticulatum*	Turkey	Wild	Wild
Kayat 077	*Cicer reticulatum*	Turkey	Wild	Wild
Kesen 075	*Cicer reticulatum*	Turkey	Wild	Wild
Oyali 084	*Cicer reticulatum*	Turkey	Wild	Wild
Oyali 111	*Cicer reticulatum*	Turkey	Wild	Wild
Sarik 067	*Cicer reticulatum*	Turkey	Wild	Wild
Savur 063	*Cicer reticulatum*	Turkey	Wild	Wild
Sirna 060	*Cicer reticulatum*	Turkey	Wild	Wild

### Experimental Design

All accessions were grown in a shade house at Fairchild Tropical Botanic Garden in Coral Gables, Florida, from December 2016 to March 2017. Average day and night temperature during this period ranged between 27 and 16°C, and average monthly rainfall was 5 cm (+/−0.75) (USclimatedata.com, 2020). Seeds of each accession were planted in 11-L pots containing 8 L of a mixture of sand and coconut coir. This mixture was used as a planting media to minimize the nitrogen present before preparation. Plants were watered every 48 h by an automatic sprinkler system.

Eight replicates of each accession were subjected to two different nitrogen treatments: 1 ppm (2.362 mg N source/L planting media) and 100 ppm (238506.2 mg/L). ESN Polymer Coated Urea (Agrium U.S. Inc.), a slow-release nitrogen pellet, was used as the nitrogen source. These treatments were chosen to represent generally nitrogen poor conditions in the wild, and the nitrogen rich conditions that may be found in an agricultural field setting, respectively (Keen, 2020). To make sure other nutrients were not limiting for chickpea growth, all pots received 2.40 mg/L phosphorus (P) as Al(PO_3_)_3_, 470.8 mg/L calcium (Ca) as CaSO_4_·2H_2_O, 507.8 mg/L magnesium (Mg) as MgSO_4_·7H_2_O, 2.598 mg/L copper (Cu) as CuSO_4_·5H_2_O, 5.401 mg/L zinc as ZnSO_4_·7H_2_O, 22.96 mg/L manganese (Mn) as MnSO_4_·H_2_O, 2.499 mg/L boron (B) as Na_2_B_4_O_7_·10H_2_O[/], and 0.119 mg/L molybdenum (Mo) as Na_2_MoO_4_·2H_2_O. Plants were grown in the absence of rhizobial symbionts, as evidence suggests that wild and cultivated chickpea differ in symbiont preference (Greenlon et al., unpublished; Greenlon et al., 2019), and symbionts differ in their tolerance of different soils (Alford et al., unpublished; Greenlon et al., 2019). All pots were randomly arranged in a grid in the shade house.

### Gas-Exchange

We measure instantaneous rates of gas exchange to estimate key aboveground traits related to carbon gain, water conservation, and its impacts on nutrient turnover. Gas-exchange measurements were performed on mature leaflets for 6–8 individuals per genotype using the LI-6400 infrared gas analyzer (Li-6400, Li-Cor Inc., NE, United States). Chamber conditions were set to 1,300 μmol PAR and CO_2_ concentration of 400 ppm. The block temperature was set to 28°C achieving an average leaf temperature of 28.91°C (+/− 1.35°C) and a cuvette vapor pressure deficit (VPD) of 1.38 kPa (+/− 0.37 kPa). After gas exchange rates had stabilized (≥6 min), net photosynthetic rates (A_N_) and stomatal conductance (g_S_) were recorded. The leaf area was corrected using digital photographs of the leaf material that was inside the chamber using ImageJ (Wayne Rasband/NIH, Bethesda, MD, United States). Gas-exchange measurements were taken between 800 and 1,300 h.

### Stable Isotope Chemistry

We measured stable isotopes of carbon and nitrogen to gain an integrated lifetime estimate of water relations and nutrient movement to complement instantaneous water use measurements. The leaflets used for gas-exchange were cut and digitally photographed in the field (for later analysis of specific leaf area) and then placed into coin envelopes and stored in a drying oven at 75°C for at least 72 h before being weighed. Leaflet area was calculated in ImageJ (Wayne Rasband/NIH, Bethesda, MD, Unites States), and specific leaf area (SLA) was calculated from the ratio of fresh area (cm^2^) and dry mass (g). The dried samples were then run through a Carlo Erba NC2500 elemental analyzer (CE Instruments Ltd., England, United Kingdom) in tandem with a Thermo Delta V Stable Isotope Mass Spectrometer (Thermo Fisher Scientific Inc., Waltham, MA, United States) at the Cornell University Isotope Lab (COIL) to measure elemental chemistry, i.e., δ13C and %N. Leaf chlorophyll investments were estimated using a Photosynthesis MultispeQ V1.0 (East Lansing, MI). Carbon isotope chemistry was used to estimate water-use-efficiency (δ13C), and total leaf carbon and nitrogen content were used to calculate C/N ratios, photosynthetic nitrogen use efficiency (PNUE), and nitrogen investment into chlorophyll (i.e., estimated by the ratio of leaf nitrogen and chlorophyll index).

### Root and Canopy Morphology

We harvested whole plants to examine plant allocation to above and belowground tissues. Aboveground and belowground plant biomass was harvested 12 weeks after sowing. Aboveground biomass was defined as all living biomass above the soil. A subset of wild and domesticated replicates of each accession for each treatment (1 and 100 ppm) was randomly selected for leaf area measurements. All leaves of selected plants were removed, laminated, and scanned at 1200 dpi using an Epson Perfection V700 scanner (Epson America, Long Beach CA). Lamination prevented folding of leaves during scanning and allowed more measurements to be taken by slowing down wilting. The total canopy area was used to scale leaf level gas exchange measurements to canopy level photosynthesis. For the remaining plants, aboveground biomass was placed in a drying oven for 24 h, after which the dry mass was recorded using an analytical balance (Mettler Toledo ME103TE, Columbus, OH, United States). All belowground biomass was carefully separated from the soil, cleaned with deionized water, and scanned for image analysis. The samples were then dried and weighed as described above. The image analysis system, WinRHIZO (version *Arabidopsis*) was used to calculate root length (i.e., RL), average root diameter (i.e., RD), root surface area (i.e., RSA), root volume (i.e., RV), and leaf area (i.e., LA) from root and leaf scans (Regent Instruments, Quebec City, QC, Canada). Specific root length and root density were calculated by dividing root length (cm) by belowground biomass (g) and belowground biomass (g) by root volume (cm^3^), respectively.

### Data Analysis

A nested linear mixed model (LMM) was used to test for significant differences for all measurements between nitrogen treatments (1 and 100 ppm), history (wild or domesticated), and accessions (i.e., ecotype). Treatment was used as a fixed factor, while accession, a random factor, was nested into history, a fixed factor, making the combined term random. Furthermore, a Tukey’s HSD test with a Bonferroni correction was used for pairwise comparisons. Furthermore, to test if ecotype had a significant effect, we calculated the value of *p* of ecotype through the *Ranova* function in the *Lmertest* package in R (Kuznetsova et al., 2017).

To understand if responses to soil nitrogen are dependent on domestication history, we examined the history by treatment interactions of our LMM for all traits (Bates et al., 2015; Harrel, 2015). To better understand the intensity of trait responses, we further calculated the relative distance plasticity index (RDPI) for traits with significant history by treatment interactions (Valladares et al., 2006). Specifically, we calculated RDPI and tested for differences between domesticated and wild chickpea for six variables: specific root length, root density, water-use efficiency, aboveground biomass, canopy level photosynthesis, leaf level photosynthesis, and stomatal conductance using the “*Plasticity*” R package (Ameztegui, 2017). Lastly, we correlated three independent variables: specific root length (x^lambda-transformed), root density (log-transformed), and water-use efficiency (x^lambda-transformed), against aboveground biomass (log-transformed) to test whether plant plasticity may affect plant fitness. For each correlation, we calculated the Pearson correlation coefficient and determined its significance using a *Psych* R package (Revelle, 2019). We used plant biomass as a fitness indicator rather than seed set because we harvested before flowering to capture intact root systems. Additionally, seed set and plant biomass are often correlated, making it an appropriate fitness indicator (e.g., von Wettberg et al., 2008). All statistical analyses were performed in R (R Core Team, 2019).

## Results

### Adaptation to the Low Nitrogen Environment

**Figure 1:**
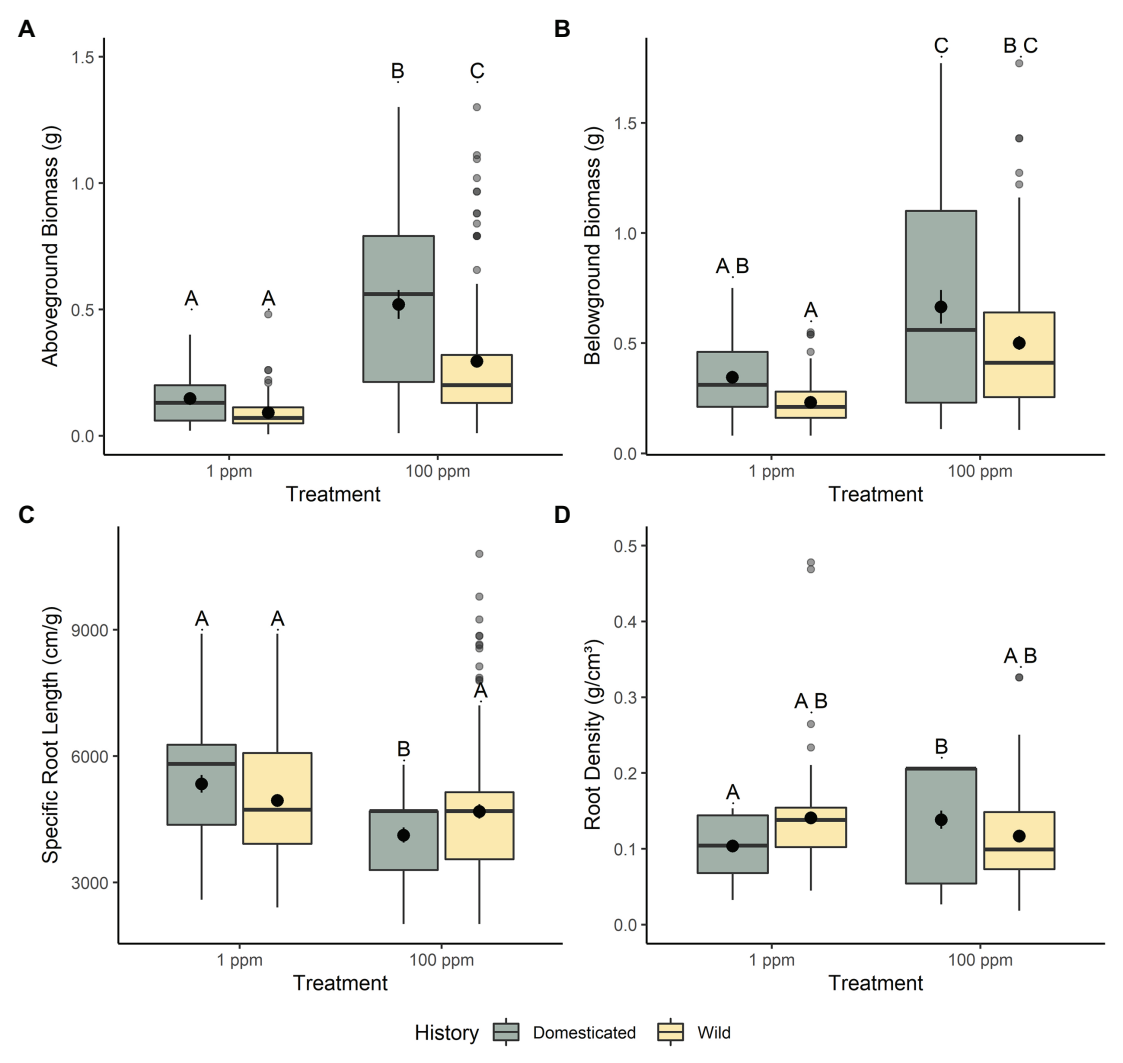
The response of chickpea morphology to increased nitrogen availability. **(A)** Aboveground biomass, **(B)** belowground biomass, **(C)** specific root length (SRL), and **(D)** root density. Domesticated (yellow) and wild (green) chickpea accessions are grouped. Different letters indicate statistically significant differences, *p* < 0.05 (Tukey’s HSD test).

Domesticated and wild chickpea displayed similar adaptations to the lower nitrogen environment (1 ppm) with only a handful of traits being significantly different between these groups. All belowground morphological measurements between domesticated and wild chickpea were non-significant; belowground biomass (*t*_46_ = 1.763, *p* = 0.507; Figure 1B), root length (*t*_49_ = 1.870, *p* = 0.404), root density (*t*_59_ = −1.940, *p* = 0.343; Figure 1D), average root diameter (*t*_53_ = 1.460, *p* = 0.902), root volume (*t*_54_ = 1.128, *p* = 1.000), SRL (*t*_87_ = 1.145, *p* = 1.000; Figure 1C), and root surface area (*t*_43_ = 1.903, *p* = 0.383).

**Figure 2:**
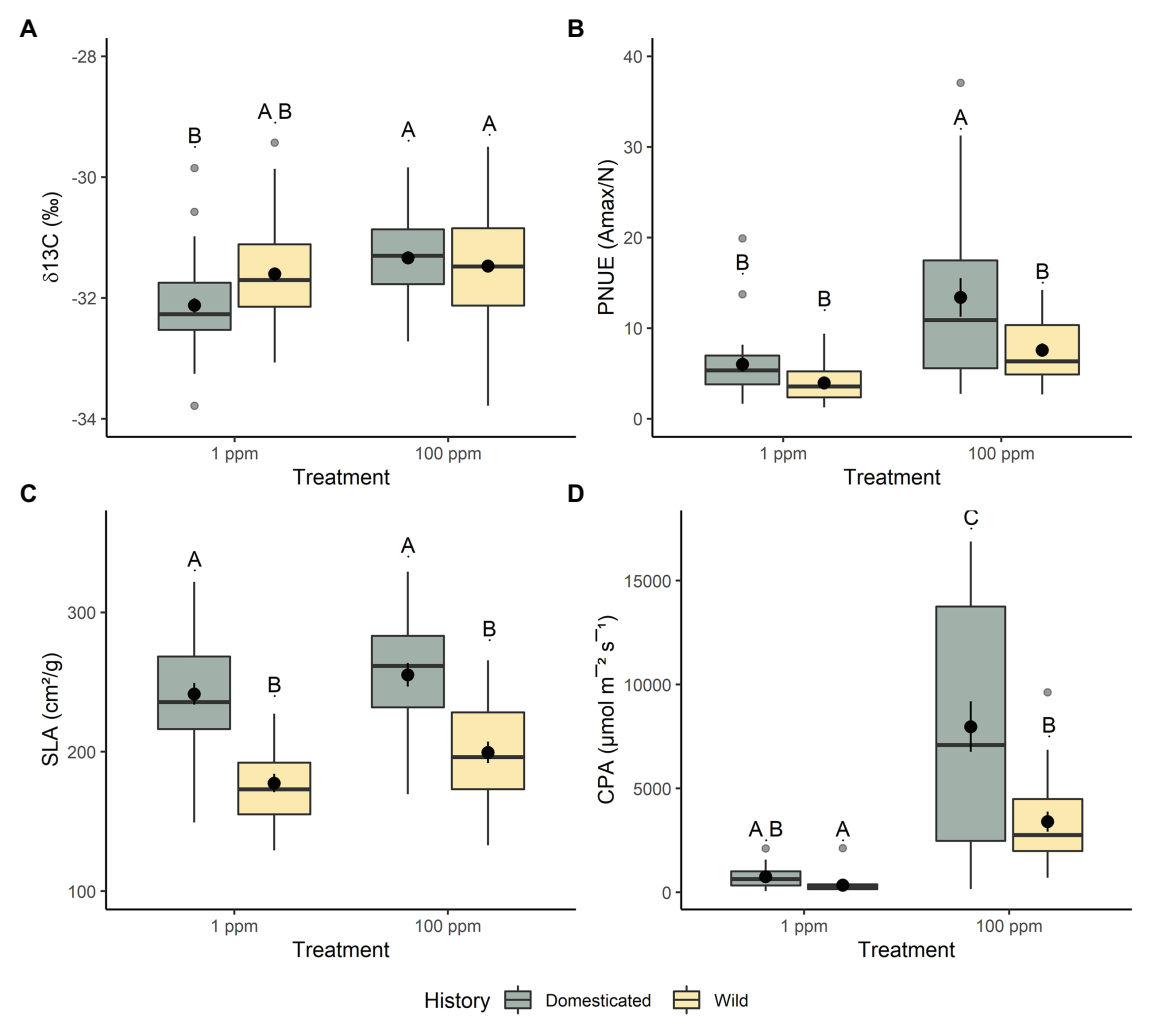
The response of chickpea morphology to increased nitrogen availability. **(A)** Water-use efficiency (δ13C), **(B)** photosynthetic nitrogen-use efficiency (PNUE), **(C)** specific leaf area (SLA), and **(D)** canopy photosynthesis (CPA). Domesticated (yellow) and wild (green) chickpea accessions are grouped. Different letters indicate statistically significant differences, *p* < 0.05 (Tukey’s HSD test).

This trend continued in regard to aboveground traits, we found very few differences between domesticated and wild chickpea in low nitrogen conditions with respect to aboveground biomass (*t*_56_ = 1.054, *p* = 1.000; Figure 1A), water-use efficiency (*t*_42_ = −2.397, *p* = 0.127; Figure 2A), %N (*t*_63_ = 1.172, *p* = 1.000), %C (*t*_85_ = 0.294, *p* = 1.000), chlorophyll content index (*t*_52_ = 1.247, *p* = 1.000), nitrogen investment into chlorophyll (*t*_48_ = −0.581, *p* = 1.000), stomatal conductance (*t*_144_ = −3.236, *p* = 0.009), canopy photosynthetic rates (*t*_27_ = 0.237, *p* = 1.000; Figure 2D), photosynthetic nitrogen use efficiency (per area *t*_32_ = 0.106, *p* = 1.000; per mass *t*_35_ = 0.697, *p* = 1.000; Figure 2B), and leaf level photosynthetic rate (photosynthetic rate per area *t*_27_ = 0.549, *p* = 1.000; leaf level photosynthetic rate per mass *t*_30_ = 0.871, *p* = 1.000). However, several other aboveground functional traits varied significantly between wild and domesticated chickpea within the low nitrogen environment, with wild chickpea displaying lower specific leaf area (*t*_41_ = 5.608, *p* = <0.001; Figure 2C) and stomatal conductance (*t*_144_ = −3.236, *p* = 0.009), and higher carbon to nitrogen ratio (*t*_292_ = −3.909, *p* = 0.001) than domesticated chickpea.

### Adaptation to the High Nitrogen Environment

Domesticated and wild chickpea displayed differential adaptation to the higher nitrogen environment (100 ppm), with several belowground and aboveground traits being significantly different between these groups. Two belowground morphological traits between domesticated and wild chickpea were significantly different from each other, indicating differences in belowground adaptation to high nitrogen conditions. Domesticated chickpea exhibited higher root volume (*t*_51_ = 3.062, *p* = 0.021) and lower SRL (*t*_77_ = −2.924, *p* = 0.0271; Figure 1C) than wild chickpea. Similar to the low nitrogen environment, domesticated and wild chickpea exhibited comparable belowground biomass (*t*_45_ = 2.215, *p* = 0.192: Figure 1B), root length (*t*_47_ = 1.267, *p* = 1.000), root density (*t*_55_ = 2.566, *p* = 0.0782; Figure 1D), average root diameter (*t*_50_ = 2.517, *p* = 0.090), and root surface area (*t*_43_ = 2.425, *p* = 0.118).

Additionally, several aboveground functional traits were significantly different between domesticated and wild chickpea within the higher nitrogen level (100 ppm), with domestic chickpea exhibiting greater aboveground biomass (*t*_53_ = 4.361, *p* = <0.001; Figure 1A), leaf level photosynthetic rate (maximum photosynthetic rate per area *t*_26_ = 3.127, *p* = 0.026; maximum photosynthetic rate per mass *t*_29_ = 4.539, *p* = <0.001), canopy photosynthetic rates (*t*_26_ = 3.605, *p* = 0.008; Figure 2D), photosynthetic nitrogen use efficiency per mass (*t*_35_ = 2.961, *p* = 0.033; Figure 2B), and specific leaf area (*t*_40_ = 4.949, *p* = <0.001; Figure 2C). Domesticated and wild chickpea did not differ in %N (*t*_59_ = 1.343, *p* = 1.000), %C (*t*_76_ = 1.083, *p* = 1.000), chlorophyll content index (*t*_49_ = 0.787, *p* = 1.000), nitrogen investment into chlorophyll (*t*_46_ = 0.352, *p* = 1.000), photosynthetic nitrogen use efficiency per area (*t*_32_ = 1.976, *p* = 3.423), water-use efficiency (*t*_40_ = 1.187, *p* = 1.000; Figure 2A), leaf C/N ratio (*t*_59_ = 0.114, *p* = 1.000), and stomatal conductance (t_107_ = −1.070, *p* = 1.000) within the higher soil nitrogen treatment (100 ppm).

### Phenotypic Plasticity: Phenotypic Response to Nitrogen Availability

Significant interactions between nitrogen level and domestication history revealed differences between wild and domesticated chickpea phenotypic response to nitrogen availability for several belowground and aboveground traits. For belowground traits, root density (*f*_1,279_ = 14.849, *p* = <0.001; Figure 1D) and SRL (*f*_1, 296_ = 8.719, *p* = 0.003; Figure 1C) were significantly different between the two groups. As nitrogen levels increased, domesticated chickpea (*t*_279_ = −3.553, *p* = 0.003) significantly increased root density, while root density for wild chickpea (*t*_279_ = 1.545, *p* = 0.741) remained relatively consistent, illustrating a canalized response (Figure 1D). Additionally, domesticated chickpea exhibited significantly reduced SRL as nitrogen level increased (*t*_284_ = 3.466, *p* = 0.004), while SRL for wild chickpea remained broadly consistent between nitrogen environments (*t*_283_ = 0.020, *p* = 1.000) (Figure 1C). These results were corroborated by our analyses of RDPI, an index that quantifies phenotypic plasticity (Valladares et al., 2006). RDPI revealed that domesticated chickpea had significantly higher plasticity for SRL (*t*_2122_ = 4.273, *p* = <0.001) and root density (*t*_2068_ = 19.059, *p* = <0.001) than wild chickpea.

Moreover, significant interactions between nitrogen level and domestication history were also present for several aboveground traits, including aboveground biomass (*f*_275_ = 8.600, *p* = 0.004; Figure 1A), water use efficiency (*f*_273_ = 16.901, *p* = <0.001; Figure 2A), C/N ratio (t_276_ = 11.471, *p* = <0.001), canopy level photosynthesis (*f*_76_ = 11.179, *p* = 0.001; Figure 2D), leaf level photosynthesis (per area, *f*_76_ = 6.462, *p* = 0.013; per mass, *f*_76_ = 11.321, *p* = 0.001), PNUE (per area *t*_29_ = 8.599, *p* = <0.001; per mass *f*_33_ = 8.941, *p* = <0.0.001; Figure 2B), and stomatal conductance (*f*_76_ = 4.137, *p* = 0.045). As nitrogen levels increased, both domesticated and wild chickpea increased aboveground biomass, whole canopy photosynthesis, leaf level photosynthesis, PNUE, and stomatal conductance. However, despite both groups increasing aboveground biomass and PNUE in response to higher nitrogen presence, domesticated chickpea exhibited higher plasticity for aboveground biomass (*t*_2232_ = 11.411, *p* = <0.001) and PNUE (per area *t*_937_ = 3.99, *p* = <0.001; per mass *t*_941_ = 3.806, *p* = <0.001) with significantly higher RDPI. On the other hand, wild chickpea had significantly higher RDPI for gas exchange traits including stomatal conductance (*t*_905_ = −4.144, *p* = <0.001), leaf level photosynthesis (photosynthetic rate per area *t*_946_ = −1.608, *p* = 0.109; photosynthetic rate per mass *t*_944_ = −2.883, *p* = 0.004), and canopy photosynthesis (*t*_938_ = −4.169, *p* = <0.001).

As nitrogen levels increased, both domesticated (*t*_279_ = 8.021, *p* = <0.001) and wild chickpea (*t*_278_ = 19.302, *p* = <0.001) significantly decreased their C/N ratio. However, despite both groups decreasing C/N ratio, wild chickpea displayed a higher phenotypic response to nitrogen presence by exhibiting a greater RDPI (*t*_2105_ = −9.943, *p* = <0.001) than domesticated chickpea. Additionally, as nitrogen levels increased, wild chickpea exhibited a canalized response with no significant change in water-use efficiency (*t*_276_ = −1.272, *p* = 1.000), whereas domesticated chickpea increased water-use efficiency (*t*_276_ = −5.612, *p* = <0.001). RDPI analyses also concluded that domesticated chickpea had a significantly higher plasticity for water-use-efficiency (*t*_1959_ = 9.794, *p* = <0.001), but this was driven by a decreased efficiency in the low soil nitrogen environment. Surprisingly, both wild (*t*_83_ = −1.282, *p* = 0.576) and domesticated (*t*_83_ = −1.937, *p* = 0.221) chickpea displayed a canalized response in regard to SLA.

### Substantial Variation by Ecotype

**Figure 3:**
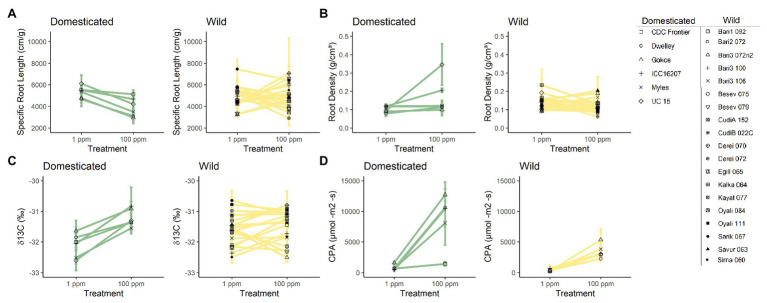
Trait means of individual chickpea accessions in low (1 ppm) and high nitrogen environments (100 ppm). **(A)** Specific root length (SRL), **(B)** root density, **(C)** water-use efficiency (δ13C), and **(D)** canopy photosynthesis (CPA). Domesticated (yellow) and wild (green) chickpea accessions are grouped. Error bars denote standard errors.

In addition to domestication history having a significant effect on chickpea’s capacity for plasticity in response to nitrogen availability, our results revealed that ecotypes also greatly varied in their response to nitrogen availability. This was determined by an overall significant random term in the linear mixed model (Figure 3). We found significant variation among ecotypes for the following belowground traits: belowground biomass [χ^2^ (1) = 17.092, *p* = <0.001], root density [χ^2^ (1) = 4.396, *p* = 0.036; Figure 3B], root length [χ^2^ (1) = 9.987, *p* = 0.002], average root diameter density [χ^2^ (1) = 8.619, *p* = 0.003], root volume [χ^2^ (1) = 7.598, *p* = 0.005], root surface area [χ^2^ (1) = 23.037, *p* = <0.001], and SRL [χ^2^ (1) = 5.298, *p* = 0.022; Figure 3A]. Additionally, a significant response variation for ecotype was found for several aboveground traits including, aboveground biomass [χ^2^ (1) = 5.506, *p* = 0.019], whole canopy photosynthesis [χ^2^ (1) = 3.957, *p* = 0.047; Figure 3D], leaf level photosynthetic rate [per area χ^2^ (1) = 4.203, *p* = 0.040], water-use efficiency [χ^2^ (1) = 26.821, *p* = <0.001; Figure 3C], chlorophyll content index [χ^2^ (1) = 9.428, *p* = 0.002], and nitrogen investment into chlorophyll [χ^2^ (1) = 12.109, *p* = <0.001].

### Phenotypic Plasticity: Relationships to Indicators of Plant Fitness

Plant size (i.e., aboveground or belowground biomass) and measures of plant plasticity were negatively correlated across treatments and history (Supplementary Figure S1). There was a significant overall negative correlation between plasticity in SRL and aboveground plant biomass (*t*_294_ = −4.838, *p* = <0.001, *r* = −0.272; Supplementary Figure S1b) and between water-use efficiency and aboveground plant biomass (*t*_294_ = −2.432, *p* = 0.016, *r* = −0.141; Supplementary Figure S1a). Root density and aboveground biomass were not significantly correlated overall (*t*_294_ = −1.298, *p* = 0.196, *r* = −0.075). The negative correlation for SRL and aboveground plant biomass held true for both wild (*t*_212 =_ − 2.321 *p* = 0.021, *r* = −0.157) and domesticated chickpea (*t*_80_ = −5.563, *p* = <0.001, *r* = −0.528) across treatments. However, for water-use efficiency and aboveground biomass, domesticated and wild chickpea differed in their correlations; with wild chickpea (*t*_212 =_ − 0.513 *p* = 0.609, *r* = −0.035; Supplementary Figure S1c) having a non-significant correlation while domesticated chickpea having a strong negative correlation (*t*_80 =_ − 4.168 *p* = <0.001, *r* = −0.422; Supplementary Figure S1d) across chickpea.

## Discussion

The overall objectives in our study were to determine (1) if wild chickpea performed better under lower nitrogen conditions and (2) if domesticated chickpea had reduced phenotypic plasticity. Our results revealed that wild and domesticated chickpea had similar phenotypes in the low nitrogen environment for both belowground and aboveground traits, indicating that the domestication has not affected the chickpea’s response to low nitrogen conditions in the absence of rhizobia. However, wild chickpea did display phenotypes consistent with water conservation, by exhibiting lower specific leaf area and stomatal conductance, adaptations consistent with one of our core hypotheses. At higher nitrogen concentrations, wild and domesticated chickpea differed in respect to many below and aboveground phenotypes, with domesticated chickpea consistently displaying phenotypes adapted to high resource conditions. This is not surprising as thousands of years of cultivation and breeding has most likely adapted domesticated chickpea, like most crops, to high resource conditions (e.g., Bogaard et al., 2013; Brown, 2018). Furthermore, significant two-way interactions between nitrogen concentration and history (wild vs. cultivated) for SRL, root density, aboveground biomass, water-use-efficiency, C/N ratio, canopy level photosynthesis, leaf level photosynthesis, and stomatal conductance demonstrated that wild and domesticated chickpea exhibited differences in their responses to nitrogen levels. Our RDPI results, surprisingly, suggest that the wild chickpea had a canalized response for SRL, root density, and greater water-use efficiency and limited phenotypic plasticity for aboveground biomass but greater phenotypic plasticity for stomatal conductance, canopy photosynthesis, leaf level photosynthesis, and C/N ratio. Both cultivated and wild chickpea exhibited a canalized response for SLA, but SLA was consistently higher for domesticated chickpea at both nitrogen treatments, indicating this trait may be adaptive for nutrient rich environments.

The lower plasticity of some traits in wild chickpea is primarily explained by the substantial ecotypic variation within the wild germplasm, which lowered the average phenotypic response to increased nitrogen. The substantial ecotypic variation in wild chickpea is not surprising as ecotypes originate from different environmental conditions (von Wettberg et al., 2018). However, the lack of plasticity for some ecotypes, yet similar performance and resource use efficiency in low nutrient conditions is surprising, as these are potential mechanisms to increase plant survival in natural environments (reviewed in Ghalambor et al., 2007; Hauvermale and Sanad, 2019). Specifically, root plasticity is beneficial for wild plants due to heterogeneous nutrient distribution and limiting nutrients found in natural habitats when compared to agroecosystems (Paz-González et al., 2000; Bennett et al., 2005). For instance, the distribution of inorganic nitrogen was found to be homogenous in agricultural top-soil, (Jackson and Bloom, 1990), while nutrient distribution varied significantly in natural sagebrush steppe-habitat (Jackson and Caldwell, 1993) and tropical forests (John et al., 2007). Furthermore, domesticated crops such as barley (Grossman and Rice, 2012), cassava (Ménard et al., 2013), and soybeans (Kiers et al., 2007) have undergone a reduction in phenotypic plasticity, which is believed to be due to a reduction in genetic diversity driven by agronomic selection (Sadras, 2007) or continuous selection in a more homogenous agricultural environment.

As expected, average aboveground and belowground biomass increased with higher nitrogen levels for both domesticated and wild chickpea. However, for several wild ecotypes, aboveground and belowground biomass decreased or remained relatively the same in higher nitrogen conditions, indicating limited phenotypic plasticity for these traits to nitrogen availability. These results are surprising, as they contrast against previous results comparing plasticity in aboveground biomass to nutrient availability in domesticated and wild: chard (*Beta vulgaris* L.), cabbage (*Brassica oleracea* DC.), sunflower (*Helianthus annuus* L.), tomato *(Solanum lycopersicum* L.), durum wheat (*Triticum durum* Desf.), maize (*Zea mays* L.), and pea (*Pisum sativum* L.; Matesanz and Milla, 2018). Differences between our results and previous findings could stem from the number of accessions used in each study. The limited number of accessions used in previous studies likely was not sufficient to adequately capture the phenotypic variation or plasticity present in each crop or wild relative (Krieg et al., 2017).

As nitrogen availability increased, domesticated chickpea ecotypes reacted uniformly with decreased SRL and increased root density. These results indicate that the domesticated chickpea has increased root diameter and decreased root length, a physiological response to higher nitrogen presence that has been shown in other studies (Callaway et al., 2003; von Wettberg and Weiner, 2003). Low SRL and high root density are optimum root phenotypes for plants in nutrient-rich soil environments, as these phenotypes are believed to be most efficient when nutrients are abundant (Reich, 2014; Kong et al., 2019). Conversely, on average, SRL and root density remained relatively unchanged for wild chickpea in both nitrogen treatments and were not significantly different. However, when taking into account how individual wild ecotypes reacted to increased nitrogen availability, we observed variation among ecotypes in phenotypically plastic responses to nitrogen. With respect to SRL and root density, wild ecotypes decreased, increased, or remained constant in response to nitrogen availability. These results were surprising as wild bean ecotypes on average have greater SRL and root density than domesticated ecotypes (Pérez-Jaramillo et al., 2017). Greater SRL has been hypothesized to provide higher efficiency of nutrient search and uptake, a beneficial phenotype for nutrient “foraging” in nutrient heterogeneous environments.

Leaf measurements such as leaf level photosynthesis and stomatal conductance, and canopy level photosynthesis were consistently greater for domesticated chickpea at both nitrogen levels; however, domestication history was not statistically significant while nitrogen level was (Supplementary Table S1). This is likely due to the cultivated varieties having been selected under higher fertility agricultural conditions when compared to those experienced by wild ecotypes. Domesticated chickpea showed a similar general increase in water-use-efficiency (less negative δ13C), %N in leaves, chlorophyll content, and PNUE to increased nitrogen availability, an expected response to nutrient-rich environments (Matesanz and Milla, 2018). When focusing on ecotypic variation, wild chickpea ecotypes did not respond consistently to increased nitrogen level in regard to water-use-efficiency (δ13C). However, at lower nitrogen conditions, wild ecotypes displayed similar nitrogen and water use phenotypes, as might be expected from adaptation to low-nitrogen conditions of the native range of wild chickpea in Southeastern Turkey. One of the few measurements that was primarily influenced by domestication history was SLA, which was not affected by increased nitrogen presence. Domesticated chickpea ecotypes had consistently higher SLA than wild chickpea (Figure 2; Supplementary Table S1), perhaps as an indirect consequence of selection in domesticated chickpea for early growth and plant maturity, as evidenced by early phenology of domesticated chickpea (Ortega et al., 2019). Additionally, domesticated chickpea had a slight increase in leaf %C relative to wild ecotypes, but this was not significant.

An important factor impacting our results is that we performed our study in the absence of symbiotic rhizobia. During root morphology measurement, nodules were rarely found. Sterile soil was an experimental necessity, as wild and cultivated chickpea differ in their preferred rhizobia and have substantial interactions with soil substrate (Cook et al., unpublished; Greenlon et al., 2019), adding multiple rhizobial strains would make the experiment too complicated to dissect a signal of response to nitrogen fertility. An experiment without rhizobia is a realistic scenario for cases when a crop is grown in new soil or in a soil that has not had chickpea for over several years prior. We suspect that wild relatives with short dispersal distances may have a greater chance to encounter nearby co-adapted symbionts than their cultivated relatives (Greenlon et al., 2019). When moved beyond their native range or grown in soils lacking a compatible symbiont, wild chickpea may consequently perform more poorly under nutrient limiting conditions. However, when wild chickpea does occur in agricultural conditions, they may on average experience fertility much higher than in uncultivated habitats. It is also possible that selection may not have been sufficient to canalize responses to low nutrient availability, particularly if there is a very limited cost to plasticity for root responses to low nutrient availability. The only existing data of which we are aware of is that of Grossman and Rice (2012), who showed a loss of root plasticity in cultivated barley ecotypes. An earlier study by Kiers et al. (2007) showed that bred soybean varieties had a reduced capacity to enforce sanctions on low-performing rhizobia, although they did not examine other root traits. Conversely, in other crops, the impacts of domestication on crop functional traits remain difficult to predict, especially for belowground traits that have not been systematically studied.

## Conclusion

The potentially widespread loss of phenotypic plasticity of crops to low fertility environments as a consequence of domestication could be a concern for farmers working on degraded or marginal soils without access to expensive inputs, as well as many organic production systems. Here, we find evidence that wild and domesticated chickpea display similarly efficient responses to low nitrogen conditions, but that domestication may have led to greater capacities for plasticity in morphological and biomass related traits but may have lowered the capacity to modify physiological traits related to gas exchange and efficient water use. Moreover, when focusing on the ecotype or variety level, we found significantly more response variation for agronomic important traits, including specific root length and water-use efficiency in wild chickpea than domesticated chickpea, indicating that the wild chickpea is a repository for novel responses to nitrogen conditions. Under Green Revolution agroecological conditions, it is not uncommon for there to be such high levels of added nitrogen in the soil that it results in reduced levels of nodulation in legumes (e.g., Kiers et al., 2007). However, if such excess nitrogen is not present, the loss of phenotypic plasticity is a concern for the performance of crops in more challenging conditions. For a crop like chickpea, which is still largely produced by small-holder farmers as a low or minimal fertilizer input crop in South Asia and East Africa and that serves a critical food security role in many diets, lost phenotypic plasticity may reduce resilience against climate change. The genetic bottlenecks that arise from domestication, post-domestication divergence, and the intensive breeding for agronomic traits may have additional, inadvertent effects on unselected belowground traits (e.g., Morrell et al., 2013; Gaut et al., 2018). These inadvertent effects are one of several reasons why large collections of wild relatives with a greater range of adaptive traits or plasticity than in the cultigen are needed in breeding programs to increase the resilience of our crops within a changing global climate (Warschefsky et al., 2014; Coyne et al., 2020).

## Data Availability Statement

The raw data supporting the conclusions of this article will be made available by the authors, without undue reservation.

## Author Contributions

EW, CK, and VP conceived and designed the experiment. ED-C, EB, EM, and CK performed the experiment. EM and CK analyzed the data. EM, CK, and EW wrote the manuscript. ES provided editorial advice. ES provided editorial advice andphotosynthetic analysis resources. All authors contributed to the article and approved the submitted version.

### Conflict of Interest

The authors declare that the research was conducted in the absence of any commercial or financial relationships that could be construed as a potential conflict of interest.

## Supplementary Material

The Supplementary Material for this article can be found online at:

https://www.frontiersin.org/articles/10.3389/fgene.2020.576338/full#supplementary-material
