# Supplementary figures and images for: The Impact of Domestication on Aboveground and Belowground Trait Responses to Nitrogen Fertilization in Wild and Cultivated Genotypes of Chickpea (Cicer sp.)
Source: Front Genet. 2020 Dec 2;11:576338. doi: 10.3389/fgene.2020.576338 (PMC7738563; doi:10.3389/fgene.2020.576338)

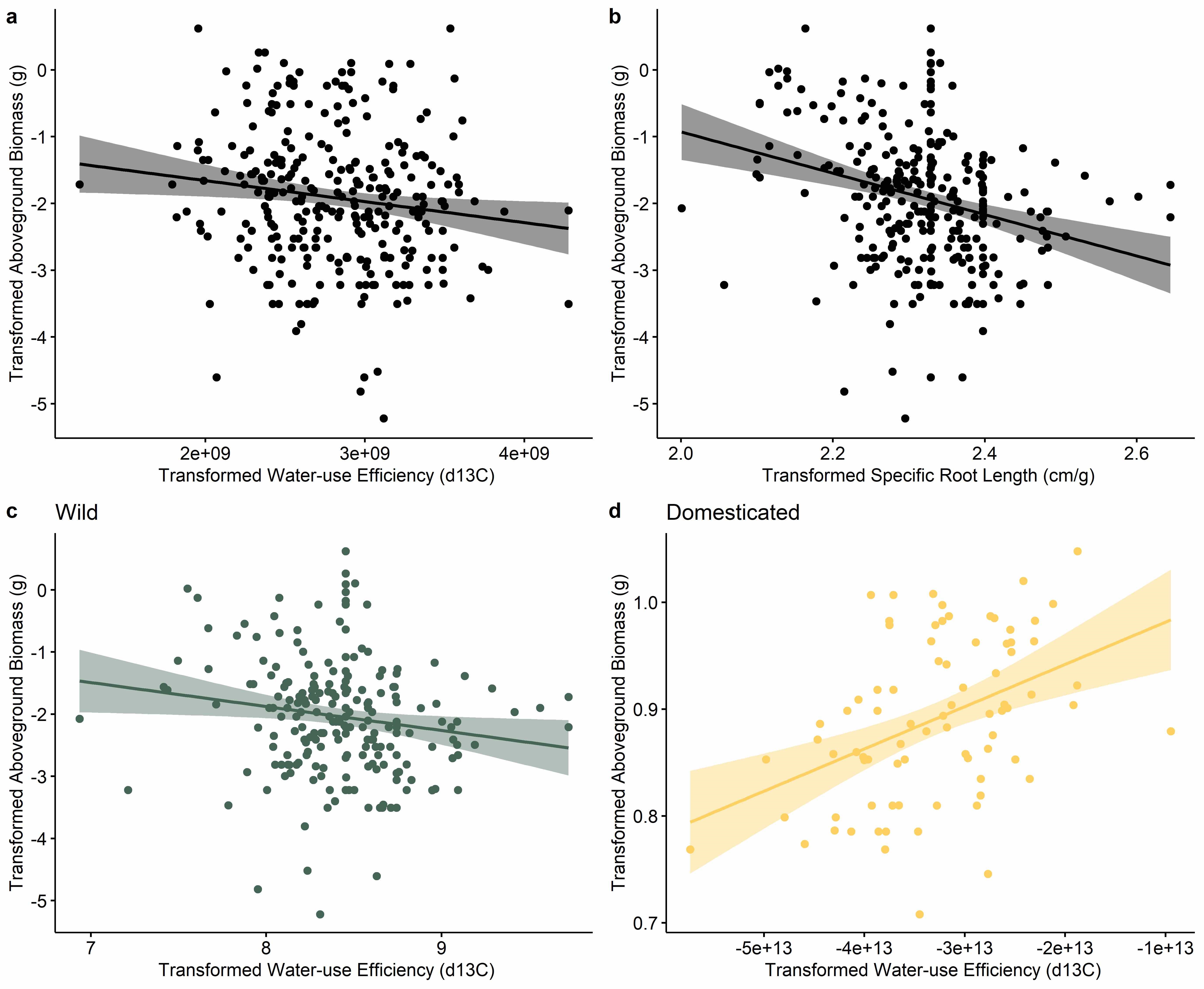

Supplement: Supplementary file 2 [file Image_1.jpeg]
